# Development of a Predictive Model for Cardiac Dysfunction in MIS-C Patients Utilizing Laboratory Biomarkers

**DOI:** 10.3390/children13020216

**Published:** 2026-02-01

**Authors:** Guliz Erdem, Brendan Galdo, Roshini S. Abraham, Allayne Stephans, Simon Lee, Jun Yasuhara, Brent Merryman, Diego Cruz Vidal, Nathan M. Money, Jennifer Colgan, Risa Bochner, Ron L. Kaplan, Erin Aldag, Thomas Graf, Steve Rust

**Affiliations:** 1 Division of Infectious Diseases, Department of Pediatrics, Nationwide Children’s Hospital, Columbus, OH 43205, USA; diego.cruz@nationwidechildrens.org; 2 Department of Information Technology Research and Innovation, Nationwide Children’s Hospital, Columbus, OH 43205, USA; brendan.galdo@nationwidechildrens.org (B.G.); steve.rust@nationwidechildrens.org (S.R.); 3 Department of Pathology and Laboratory Medicine, Nationwide Children’s Hospital, Columbus, OH 43205, USA; roshini.abraham@nationwidechildrens.org (R.S.A.); brent.merryman@nationwidechildrens.org (B.M.); 4 Section of Pediatric Hospital Medicine, Department of Pediatrics, Case Western Reserve University, Rainbow Babies and Children’s Hospital, Cleveland, OH 44111, USA; allayne.stephans@uhhospitals.org (A.S.); thomas.graf@uhhospitals.org (T.G.); 5 Department of Cardiology, Ann and Robert H. Lurie Children’s Hospital of Chicago, Northwestern University Feinberg School of Medicine, Chicago, IL 60611, USA; silee@luriechildrens.org; 6 Department of Cardiology, The Royal Children’s Hospital, Melbourne, VIC 3052, Australia; jun.yasuhara@med-ikushinkai.com; 7 Section of Pediatric Hospital Medicine, Department of Pediatrics, University of Utah School of Medicine, Primary Children’s Hospital, Salt Lake City, UT 84113, USA; nathan.money@hsc.utah.edu (N.M.M.); erin.aldag@hsc.utah.edu (E.A.); 8 Division of Emergency Medicine, Ann and Robert H. Lurie Children’s Hospital of Chicago, Northwestern University Feinberg School of Medicine, Chicago, IL 60611, USA; jcolgan@luriechildrens.org; 9 Department of Pediatrics, New York City Health and Hospitals Harlem Hospital, Columbia Vagelos College of Physicians and Surgeons, New York, NY 10032, USA; bochnerr@nychhc.org; 10 Department of Pediatrics, University of Washington School of Medicine, Seattle Children’s Hospital, Seattle, WA 98105, USA; ron.kaplan@seattlechildrens.org

**Keywords:** MIS-C, Kawasaki disease, left ventricular systolic dysfunction, data analytics

## Abstract

Background and Objectives: Early identification of cardiac dysfunction in multi-system inflammatory syndrome in children (MIS-C) is crucial for effective management. Our primary objective was to predict left ventricular systolic dysfunction (LVSD) through a multicenter collaborative assessing admission laboratory data and echocardiogram findings. Methods: Laboratory and clinical data were collected by retrospective chart review from a cohort of pediatric patients admitted and treated for MIS-C in our institutions. Laboratory data including absolute lymphocyte count, albumin, sedimentation rate, C-reactive protein, procalcitonin, d-dimer, fibrinogen, ferritin, interleukin-6 level, and lymphocyte subsets (T, B and NK quantitation, TBNK) were collected. We built a LASSO logistic regression model to predict which MIS-C patients would have left ventricular systolic dysfunction LVSD using only laboratory data obtained within the first 24 h of admission. Results: Of the 1474 MIS-C patients evaluated, 297 had LVSD. The linear kinetic analysis found differences in albumin, lymphocyte count, C-reactive proteins and fibrinogen for systolic dysfunction patients, and of these C-reactive proteins, fibrinogen and procalcitonin were more predictive earlier. The best model for coronary artery abnormalities (CAAs) performed poorly, with a mean cross-validated AUC of 0.57. The model performed well with a cross-validated AUC of 0.845. Conclusions: This model identified widely available biomarkers to successfully predict systolic dysfunction in MIS-C patients. Those at high risk of systolic dysfunction had higher peak laboratory values for C-reactive protein, fibrinogen, and procalcitonin early on. A regularized logistic regression model was validated to provide excellent discrimination for LVSD.

## 1. Introduction

Multisystem inflammatory syndrome in children (MIS-C) is a severe hyperinflammatory condition following an acute severe acute respiratory syndrome coronavirus-2 (SARS-CoV-2) infection. MIS-C was diagnosed in patients younger than 21 years old, with severe cardiovascular or multisystem clinical manifestations, laboratory evidence of inflammation, and either laboratory evidence of SARS-CoV-2 infection or epidemiologic association with COVID-19 [1,2,3,4]. The inflammatory response in MIS-C has been associated with myocarditis and initially with coronary artery changes (CAAs), similar to Kawasaki disease (KD) [1,2,3,4]. Initial studies suggested that African American children are affected disproportionately [1,2]. Recent studies suggest that unlike KD, cardiac dysfunction and CAAs resolve in MIS-C patients with prompt diagnosis and appropriate treatment [5]. However, like KD, clinicians are tasked to make the MIS-C diagnosis based on a constellation of findings in the absence of a gold standard diagnostic test. There is currently no machine learning algorithm with readily available clinical and laboratory variables distinguishing MIS-C patients at risk of developing cardiac involvement [6,7,8]. Cardiac involvement can be assessed by obtaining cardiac-specific enzymes and echocardiograms, but these may be initially normal or not readily available at the time of initial presentation. Using data from a multi-center retrospective cohort study, a machine learning algorithm was developed to predict cardiac involvement during the initial evaluation of MIS-C patients.

## 2. Materials and Methods

### 2.1. Data

We performed a multicenter retrospective cohort analysis at 6 academic urban children’s hospitals from the Pacific Northwest, Mountain West, and Midwest regions in the United States. Institutional review board approval was obtained at each institution before data collection. MIS-C patients admitted from 7 June 2020, to 2 March 2022 were included. Children who were 6 months to 20 years old who had a D-dimer or SARS-CoV-2 IgG antibody test performed between 31 March 2020, to 1 February 2022, were identified by data query. We chose these screening criteria based on initial workup practices at our institutions for presumed MIS-C. A manual chart review was performed on all subjects, and patients who were diagnosed with MIS-C by the treating provider during their hospitalization were included in the analysis. Patients evaluated for MIS-C but ultimately diagnosed with alternate conditions were excluded. No patients were excluded based on chronic medical conditions.

Demographics, clinical and laboratory information were collected from electronic chart reviews. Admission and discharge diagnoses were collected, and uncertainties about final discharge diagnoses were adjudicated among authors. Variables involving presenting symptoms and exam findings were chosen based on common symptoms and exam findings for patients with MIS-C. Historical symptoms were recorded as positive only if mentioned in the electronic health record. Laboratory tests included quantitative d-dimer (QDDIM), fibrinogen (FIBR), partial thromboplastin time, prothrombin time, international normalized ratio, lactate dehydrogenase, albumin (ALB), ferritin (FERR), troponin, B-type natriuretic peptide (BNP), complete blood cell counts, absolute lymphocyte count (ALC), erythrocyte sedimentation rate (ESR), C-reactive protein (CRP), procalcitonin (PCT), aspartate aminotransferase, alanine aminotransferase, triglyceride level, interleukin 6. Initial and repeat the echocardiogram results were collected. If there was no echocardiogram obtained, patients were assumed to have no cardiac dysfunction.

### 2.2. Participants

The included patients were MIS-C patients admitted to six children’s hospitals from 7 June 2020 to 2 March 2022 (Table 1). Children 6 months to 20 years old who had a D-dimer or SARS-CoV-2 IgG antibody test per-formed between 31 March 2020, to 1 February 2022, were identified by data query. We chose these screening criteria based on initial workup practices at our institutions for presumed MIS-C. A manual chart review was performed on all subjects, and patients who were diagnosed with MIS-C by the treating provider during their hospitalization were included in the analysis. Patients evaluated for MIS-C but ultimately diagnosed with alternate conditions were excluded. No patients were excluded based on chronic medical conditions.

### 2.3. Data Preparation

A LASSO logistic regression model was built to predict which MIS-C patients would have left ventricular systolic dysfunction (LVSD) using initial laboratory biomarkers as predictors. Patients were only included in the analysis if they were: (1) young age (typically <19 years old); (2) severe cardiovascular or multisystem clinical manifestations; (3) laboratory evidence of inflammation; and (4) either laboratory evidence of SARS-CoV-2 infection or epidemiologic association with COVID-19 (including temporal association with periods of high local COVID-19 transmission [4].Least Absolute Shrinkage and Selection Operator (LASSO) regression [9] was used to select the most predictive measures (feature selection) while simultaneously estimating the model coefficients.

### 2.4. Outcomes

ALASSO logistic regression modelwas built to predict which MIS-C patients would have left ventricular systolic dysfunction using only laboratory data obtained within the first 24 hours of admission.

### 2.5. Predictors

Each subject’s first measure of ALB, ALC, CRP, FERR, FIBR, PCT, QDDIM, ESR, interleukin 6, BNP, age and sex were considered as predictors. We transformed highly right-skewed predictors using either a square root or logarithmic (base 10) transformation if it made the marginal distribution more normal and less skewed. Following this, missing laboratory measures were imputed using the soft-impute algorithm [10]. After transformation and imputation, all variables were normalized (mean=0, standard deviation=1) during model training to ensure the amount of regularization applied to a variable’s coefficient was not influenced by a variable’s scale. 

### 2.6. Model Training and Evaluation

LASSO logistic regression models were fit using the glmnet R package v. 4.1-4 [11]. Following the internal validation of our model at Nationwide Children’s Hospital, we used a leave-one-institution-out cross-validation (CV) scheme to estimate the predictive performance of the model and optimize the LASSO penalty hyper parameters via a grid search; each model variant was trained using all but one of the institution’s data, and model performance was assessed using the predictions for the withheld institution. This was repeated until all the institutions were held out, and then the out-of-sample predictions from all institutions were combined. We selected the LASSO penalty that led to the highest overall out-of-sample performance in terms of area under the receiver operating characteristic curve (AUC).

### 2.7. Analysis

Performance was assessed using a receiver operating characteristic (ROC) curve computed using pROC R package version 1.18.0 [12,13] and positive predictive value (PPV) curve. Performance was then categorized by institution. Because the prevalence of cardiac dysfunction in MIS-C patients differed between institutions, comparisons between institutions based on prevalence-dependent measures such as PPV were not meaningful. Alternatively, we compared institution-specific performance using markedness [14] and NetPPV. Markedness (PPV+NPV-1) is the probability a condition was marked by the predictive ability of model rather than chance [14], while NetPPV, defined as (PPV − Prevalence)/(1 − Prevalence), standardizes PPV based on prevalence such that maximal performance is 1 and chance performance (randomly guessing at the rate of prevalence) is 0 regardless of prevalence. To elaborate with an example, a NetPPV of 0.5 indicates that the PPV is halfway between the baseline (performance equivalent to random chance) and the ideal outcome (where PPV equals 1). In this hypothetical, a model captures 50 % of the maximum achievable improvement in PPV. While a model with NetPPV of 0.75 indicates achievement of 75% of the maximum achievable above chance. For both markedness and NetPPV, 0 indicates the model is useless (on par with chance performance), while 1 is the best possible performance.

Knowing that laboratory markers such as interleukin 6, procalcitonin, and ferritin are not always readily available or frequently tested at some institutions, we constructed a second model where we followed an identical procedure but omitted interleukin 6, procalcitonin, and ferritin from the candidate variable list. We refer to this as the “reduced model” and the model that considered interleukin 6, procalcitonin, and ferritin the “full model”.

## 3. Results

During the study period, 297 of 1474 MIS-C patients had confirmed cardiac dysfunction via echo cardiogram. The study was one of the largest within the United States. The mean age was 7.85 years (standard deviation = 5.53 years), 46.45 percent were female, and 53.55 percent were male. In terms of race, 47.69 percent of patients were white, 29.17 percent of patients were black, 0.61 percent were American Indian, 0.81 percent were pacific islander, and the remaining 21.72 percent of patients’ races were unknown. In terms of ethnicity, 19.06 percent were Hispanic or Latino, 71.23 were documented as not Hispanic or Latino, and the remaining 9.71 percent unknown.

For the full model, the LASSO method selected 12 of 13 candidate variables as significant predictors of cardiac involvement. These variables were albumin, absolute lymphocyte count, ferritin, fibrinogen, procalcitonin, d-dimer, erythrocyte sedimentation rate, interleukin 6, age, and sex. The C-reactive protein variable was not selected. In the reduced model, after dropping interleukin 6, ferritin, and procalcitonin, the C-reactive protein variable was selected. This is likely because C-reactive protein was correlated with interleukin 6, ferritin, and procalcitonin in our data. C-reactive protein was not available in all centers during the first evaluation as a standard test. Table 1 contains the area under the for receiver operating curve for participating institutions. Table 2 contains the intercepts and estimated coefficients for each model, along with odds ratios for each predictor.

**Figure 1:**
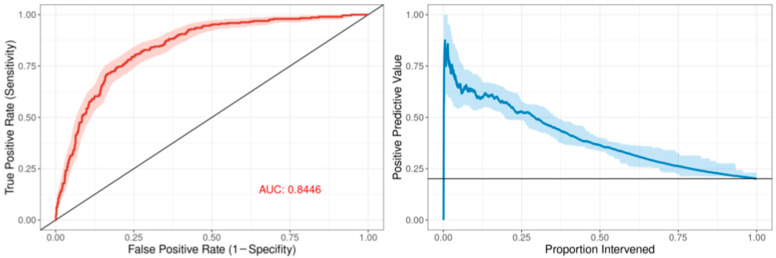
Predictive model performance: The left panel shows the receiver operating characteristic (ROC) curve in dark red comprising the mean repeated cross-validation predictions for each of the patients. The area under the curve (AUC) is denoted in red text. The 95% bootstrapped confidence band is represented by the red shaded area. The right panel shows the positive predictive value as a function of the proportion of patients intervened using the mean cross-validation predictions for each patient. The 95% bootstrapped confidence band is represented by the light-blue shaded area.

**Figure 2:**
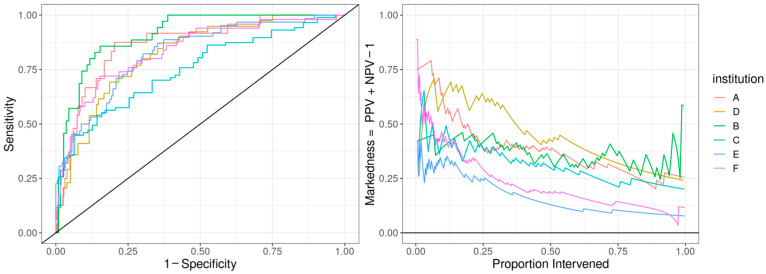
Predictive model performance by institution: The left panel shows the receiver operating characteristic (ROC) curve with the held-out performance for each institution. The right panel shows the markedness, the probability that a condition (cardiac dysfunction) is marked by the model vs. marked chance. We display markedness (PPV + NPV − 1) as opposed to PPV because the base rate of cardiac dysfunction in MIS-C patients and markedness is base-rate-independent.

**Figure 3:**
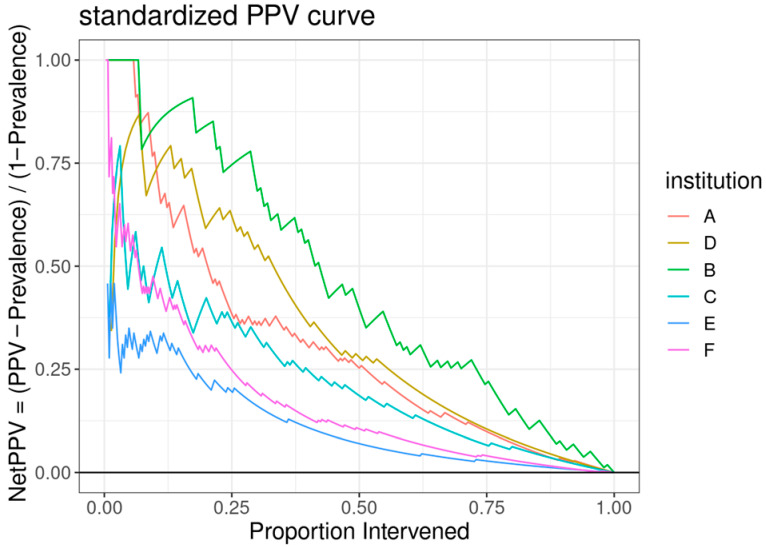
A standardized positive predictive value (PPV) curve, with the *y*-axis representing the “NetPPV” calculated as “PPV/(1 − Prevalence)” and the *x*-axis indicating the “Proportion Intervened”. Multiple curves are plotted for different institutions, each distinguished by a unique color.

The ROC curve for the best-performing model in terms of cross-validated AUC is shown in Figure 1. The model generally performed well, with an out-of-sample AUC of 0.845 for all institutions data combined. Additionally, the reduced model performed well, with only a small reduction in out-of-sample AUC, dropping from 0.845 to 0.831. We assessed the institution-specific performance in terms of AUC (Table 2, Figure 2). In terms of out-of-sample AUC, the model performed best at institution B, while performing worst at institution C. However, at low false positive rate (high specificities), the model performance on institution C data was comparable to other institutions. Looking at the markedness of each of model, we found a similar ordering of institution-specific performance, except institution B falls within the middle of pack and institution E falls to the bottom. Alternatively, when we examined the NetPPV (Figure 3), we found a similar ordering of performance as for markedness, but the model performs best at institution B. This difference is likely to due to the fact NetPPV directly considers false negatives, while markedness considers both NPV and PPV.

## 4. Discussion

Several studies published to date have reported clinical and laboratory findings that could be associated with cardiac involvement and disease severity in patients with MIS-C [4,15,16,17]. Our study demonstrates that a predictive modeling tool using machine learning may allow for timely identification of patients with the highest risk of cardiac dysfunction. Since MIS-C is a hyperinflammatory disease process, it is not surprising that markers of inflammation appeared relevant. The relationship with interleukin 6 has also been studied, showing increased levels in MIS-C [18]. In one study, interleukin 6 levels were found to be lower than what is observed in sepsis, and not discriminatory between MIS-C patients with or without features of shock [19]. Interleukin 6 levels may be useful in the context of a multi-analyte model such as ours in predicting impaired cardiac function. Recently, among inflammatory markers, presepsin has been evaluated in predicting complications of viral infections including SARS-CoV-2 [20].

Other observations with LVSD were lower absolute lymphocyte counts and worsening hypoalbuminemia. Hypoalbuminemia could be attributed to hyperinflammation and capillary leak syndrome, but many of the children who had these findings did not manifest them on admission. The worsening lymphocyte counts and albumin levels could also be dependent on the duration of symptoms at the time of the presentation. Most of our patients had symptom duration of less than three days, and even if we were not able to standardize the time from onset of illness to the clinical worsening, we were able to standardize our model from the time of their presentation.

The retrospective nature of data collection, and possible exclusion of patients with milder illness who were not diagnosed with MIS-C, were possible limitations of our multi- site study. As retrospective observational data were used, measurements were not systematically obtained across all patients at specific times. Although selected variables were found to maximize the predictive power of this model, some variables may be surrogates for other variables with causal relationships that were not selected or perhaps were not included in the data set.

To the best of our knowledge, this is the first study of its kind to utilize a combination of laboratory markers in early prediction of cardiac dysfunction among MIS-C patients. In settings where cardiac-specific tests are not readily available, our predictive model provides risk information to support effective clinical management decision-making. Obtaining repeated laboratory measurements such as serum albumin might be useful in early identification of patients at risk of deterioration and/or cardiac involvement. Although the incidence of MIS-C has decreased substantially since the start of the pandemic, cases are still being reported across the world. In 2023, CDC reported that the incidence was 0.11 cases per million person-months, representing an 80% decline compared to the peak in late 2020-early 2021, with a relative increase in cases among unvaccinated children [21]. Additionally, this approach could be used to develop risk prediction models for other hyperinflammatory conditions, such as Kawasaki disease.

## 5. Conclusions

Our model identified widely available biomarkers to successfully predict systolic dysfunction in MIS-C patients.

## Figures and Tables

**Table 1:** The area under the receiver operating curve (AUC) by the institution. The full model considered all candidate variables before variable selection, while the reduced model did not consider the less standard labs: interleukin 6, procalcitonin, and ferritin.

Institution	AUC Full	AUC Reduced
A	0.864	0.844
B	0.910	0.899
C	0.749	0.733
D	0.819	0.792
E	0.825	0.798
F	0.835	0.833

**Table 2:** Estimated model parameters as logistic regression coefficients and equivalent odds ratios. The last column denotes the percent of patients with that lab missing. The full model considered all candidate variables before variable selection, while the reduced model did not consider the less standard tests: interleukin 6, procalcitonin, and ferritin.

Variable	Coefficient (Full Model)	Odds Ratio (Full Model)	Coefficient (Reduced Model)	Odds Ratio (Reduced Model)	Unit (Before Transformation)	Missing Rate
**Intercept**	−4.74716	NA	−3.76871	NA	NA	NA
FEMALE	−0.40764	0.66522	−0.30199	0.73935	NA	0.5%
ALB	−0.13917	0.87008	−0.04326	0.95766	(g/dL)	6.2%
Log_10_(ALC)	−0.11308	0.89308	−0.52366	0.59235	cells/microL	4.4%
Log_10_(QDDIM)	−0.04381	0.95714	0.23945	1.27055	ng/dL	11.5%
sqrt(SED)	0.02516	1.02548	0.02546	1.02579	mm/hr	29.0%
Age	0.02789	1.02829	0.01076	1.01082	years	0.5%
sqrt(FIBR)	0.04201	1.04290	0.05830	1.06004	mg/dL	43.1%
Log_10_(PROCAL)	0.16349	1.17761	NA	NA	ng/ML	56.7%
**Log_10_(IL6)**	0.17639	1.19290	NA	NA	pg/mL	89.6%
**Log_10_(FERR)**	0.59057	1.80502	NA	NA	mcg/L	27.9%
**Log_10_(BNP)**	1.05006	2.85783	1.03917	2.82688	pg/mL	16.1
Log_10_(CRP)	NA	NA	0.45285	1.57279	mg/dL	4.7%

Abbreviations: ALB: albumin, ALC: absolute lymphocyte count, QDDIM: quantitative d-dimer, SED: sedimentation rate, FIBR: fibrinogen, PROCAL: procalcitonin, IL6: interleukin-6, FERR: ferritin, BNP: B-type natriuretic peptide, CRP: C-reactive protein.

## Data Availability

The data presented in this study are available on request from the corresponding author. The data are not publicly available due to privacy and ethical reasons.
